# Dr. Cyrus Edson

**Published:** 1904-01

**Authors:** 


Dr. Cyrus Edson, of New York, died of pneumonia, December 2, 1903, at Roosevelt Hospital, after a short illness, aged 46 years. He was health commissioner during a portion of the administrations of Mayor Gilroy and Mayor Strong. He worked out important problems in the health department and acquired a large practice after his retirement from office.

